# GlASS - Global Aggregation of Stream Silica

**DOI:** 10.1038/s41597-025-05937-2

**Published:** 2025-10-20

**Authors:** Kathi Jo Jankowski, Keira Johnson, Nicholas J. Lyon, Sidney A. Bush, Paul Julian, Lienne R. Sethna, Diane M. McKnight, William H. McDowell, Adam S. Wymore, Pirkko Kortelainen, Hjalmar Laudon, Ruth C. Heindel, Amanda E. Poste, Arial Shogren, Fred Worral, Luke Mosley, Pamela L. Sullivan, Joanna C. Carey

**Affiliations:** 1 U.S. Geological Survey, Upper Midwest Environmental Sciences Center, La Crosse, WI USA; 2 College of Earth, Ocean, and Atmospheric Sciences, Oregon State University, Oregon, USA; 3 National Center for Ecological Analysis and Synthesis, University of California, Santa Barbara, CA USA; 4 College of Earth Ocean and Atmospheric Sciences, Oregon State University, Corvallis, OR USA; 5 Everglades Foundation, Palmetto Bay, FL USA; 6 St. Croix Watershed Research Station, Marine on St. Croix, MN 55047 USA; 7 Department of Civil, Environmental, and Architectural Engineering, University of Colorado Boulder, Boulder, Colorado, 80309 USA; 8 Department of Natural Resources and the Environment, University of New Hampshire, Durham, NH 03824 USA; 9 Finnish Environment Institute, Helsinki, Finland; 10 Forest Ecology and Management, Swedish University of Agricultural Sciences, Umeå, Sweden; 11 Environmental Studies Program, Kenyon College, Gambier, Ohio 43022 USA; 12 Department of Arctic Ecology, Norwegian Institute for Nature Research, Tromsø, Norway & Norwegian Institute for Water Research, Oslo, Norway; 13 Department of Biological Sciences, University of Alabama, Tuscaloosa, AL 35457 USA; 14 Department of Earth Sciences, University of Durham, Durham, United Kingdom; 15 School of Agriculture, Food and Wine, The University of Adelaide, South Australia, Australia; 16 College of Earth Ocean and Atmospheric Sciences, Oregon State University, Oregon, USA; 17 Math, Analytics, Science & Technology Division. Babson College, Wellesley, MA USA

**Keywords:** Element cycles, Limnology

## Abstract

Riverine silicon (Si) plays a vital role in governing primary production, water quality, and carbon cycling. Climate and land cover change have altered how dissolved Si (DSi) is processed on land, transported to rivers, and cycled through aquatic ecosystems. The Global Aggregation of Stream Silica (GlASS) database was constructed to assess changes in river Si concentrations and fluxes, their relationship to other nutrients (nitrogen (N) and phosphorus (P)), and to evaluate mechanisms driving the availability of Si. GlASS includes concentrations of DSi, dissolved inorganic N (NO_3_, NO_x_, and NH_4_), and dissolved inorganic P (as soluble reactive P or PO_4_-P) at daily to quarterly time steps from 1963 to 2024; daily discharge; and watershed characteristics for 421 rivers spanning eight climate zones. Original data sources are cited, data quality assurance workflows are public, and input files to a common load model are provided. GlASS offers critical data to address questions about patterns, controls, and trajectories of global river Si biogeochemistry and stoichiometry.

## Background & Summary

River ecosystems fundamentally link the biogeochemical cycling of elements along the land-ocean continuum^1–3^. This link is especially true for silicon (Si), as rivers deliver >80% of annual Si loads to global oceans^4,5^. Dissolved Si (DSi) transported by rivers directly links to global weathering, nutrient, and carbon (C) cycles along the terrestrial-marine continuum, most notably through primary production by siliceous diatoms^4,6^ which represent ~20% of photosynthetically fixed CO_2_ each year^7–9^. Unlike other phytoplankton, freshwater, coastal and marine diatoms require Si in large quantities to grow. Marine diatoms typically require equal quantities of Si and N on a molar basis, whereas freshwater diatoms have greater Si requirements relative to nitrogen (N) and phosphorus (P)^6,10^. In the presence of excess N and P, Si can become limiting to diatom growth, shifting phytoplankton community composition away from diatoms towards non-siliceous algae and cyanobacteria^7,11,12^. Despite the important role of rivers in processing and supplying Si needed for diatom growth, particularly for downstream marine systems where Si is often strongly limiting, we have far less knowledge of the controls and variability in space and time of river Si exports than for other nutrients.

**Fig. 1:**
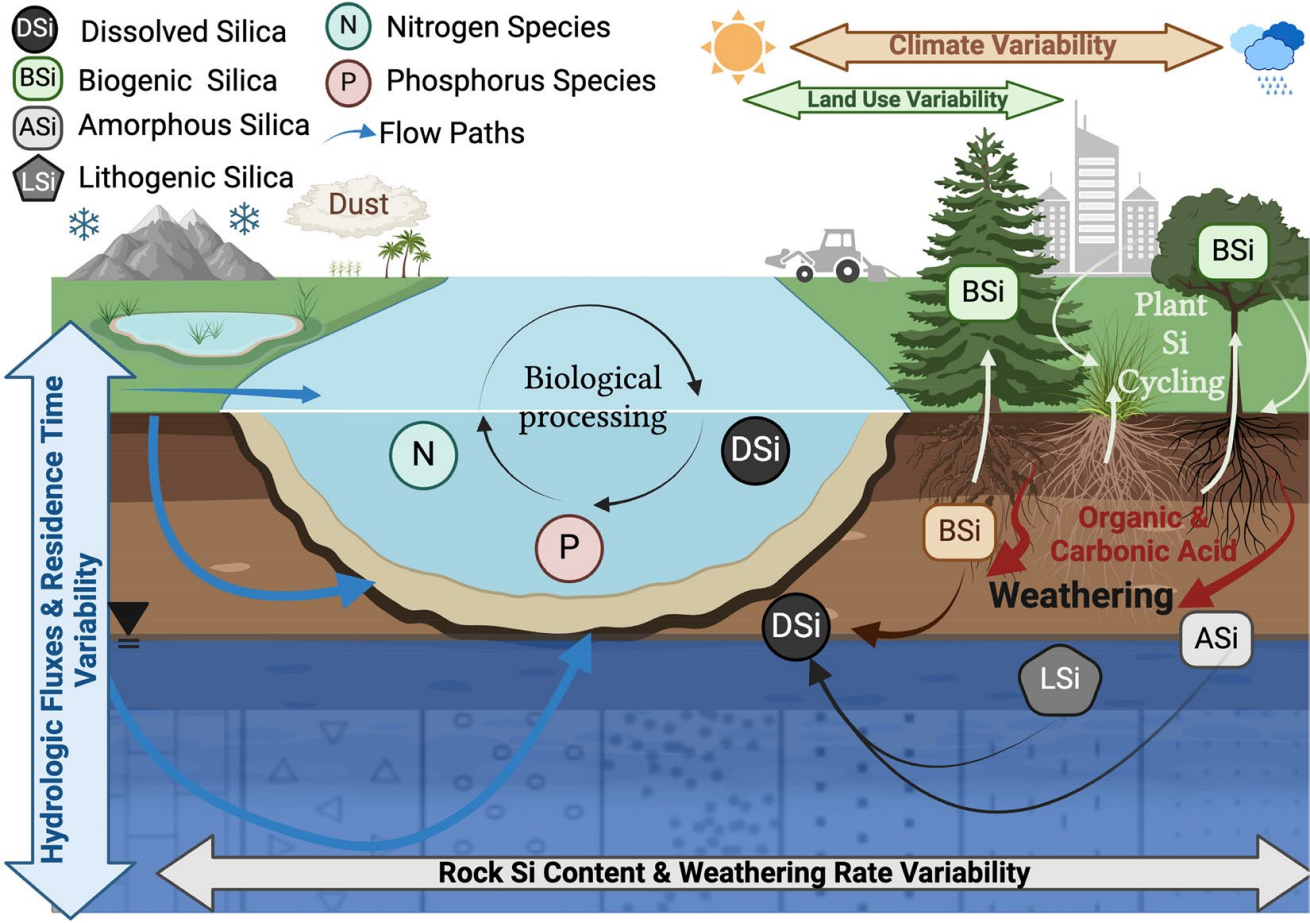
Conceptual figure of the terrestrial and aquatic controls on riverine dissolved silicon (DSi) concentrations and yields. Silica derived from geogenic processes occurs either through the breakdown of rock or soil at a specific site or through the input and eventual breakdown of dust. DSi can be taken up through biotic processes (e.g., vegetation or diatoms) or precipitated as amorphous silica. DSi is transported from land to streams across various hydrologic flow paths. The interaction of the processes and their control on DSi concentrations and fluxes is dependent on the type of underlying lithology, land cover, land use, and climate. Double headed arrows in the figure indicate a wide range of variability of a given process (from Jankowski *et al*.^17^).

The total flux of river Si exported to global oceans is controlled by complex ecological, geological, and climatic processes that vary throughout the river network (Fig. 1). With the exception of river damming, which is well known to modify river Si exports^13–15^, river Si fluxes are often assumed to be relatively stable over time, unaffected by human disturbance due to the dominant role of lithological weathering in controlling river Si exports^16^. Few examinations of changes in river Si over time have been completed over large spatial scales. Using a portion of the dataset presented here, dissolved Si (DSi) concentrations and yields were found to be changing over time, with the majority (62%) of the 60 rivers examined displaying significant increases in DSi yields over the past two decades^17^. These shifts were observed across a wide range of biome types, but most markedly in alpine and polar regions, which are particularly vulnerable to climate warming^18^. Watershed biogeochemical processes (e.g., changes in terrestrial vegetation, permafrost melt) were indicated as a driver of shifting fluxes, rather than simply changing streamflow.

In addition to investigating long-term (>20 year) changes in river DSi exports, GlASS has been used to characterize the seasonal cycles, or regimes, of river DSi concentrations^19^. Seasonal regimes are integral to understanding how river ecosystems function, as they reflect the integrated signal of hydroclimatic conditions, biological processes, and watershed characteristics, including lithology, land cover, and vegetation^20–22^. Few studies have investigated the seasonal regimes of river Si concentration across broad spatial scales, with most prior work examining temperate rivers and identifying a fairly singular pattern of a spring drawdown and an elevated winter plateau^23,24^. Using a subset of the dataset presented here, five distinct seasonal Si regimes across the Northern Hemisphere were identified, documenting how the seasonal timing of maximum and minimum concentrations varied widely among rivers^19^. Most rivers exhibited multiple regimes over time, rather than a consistent seasonal pattern. The same subset of GlASS was then used to determine the watershed-scale drivers controlling the variation in seasonal regimes^22^. Variation in seasonal regime was associated with a suite of climate- and ecosystem productivity-related factors, such as snow cover, temperature, green-up day, and evapotranspiration. Together, this work provides fundamental new insights about river Si cycling and highlights the diversity of processes controlling watershed Si cycling.

Assessing controls on river Si exports at large spatial scales requires relating spatially-extensive stream chemistry data to river flow and watershed climate, land cover/vegetation type, land use, and lithology characteristics. Although many agency, country, university and research monitoring efforts have collected Si data in rivers across the globe, these disparate datasets have not been combined into a single publicly-available database. Additionally, these data sources vary in terms of time span, sampling protocols, chemical species measured, documentation, data curation, and data accessibility.

**Fig. 2:**
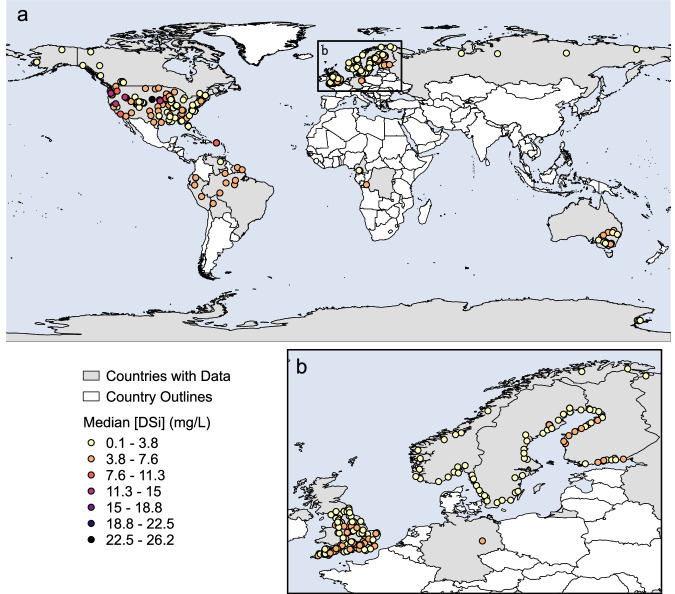
Global spatial distribution (**a**) of rivers included in GlASS. Points indicate location of discharge and chemistry measurement, and are colored by median DSi concentration. Inset box shows Scandinavia (**b**).

To overcome this shortcoming, we developed the Global Aggregation of Stream Silica (GlASS) database to harmonize DSi datasets generated using different sampling methods, levels of documentation, and conventions for naming, units, and other characteristics to address critical ecological questions at large spatial scales. To understand the dominant controls on Si we integrated several additional variables such as concentrations of other solutes (e.g., N, P), river hydrology, and watershed characteristics into the GlASS database. To achieve this goal, data from national and state level monitoring programs, the U.S. LTER Network, private research institutions, and individual researchers were combined to create a georeferenced stream Si database with over 600,000 individual nutrient chemistry observations collected across 421 rivers (Fig. 2). In addition, each stream has paired daily discharge data, which is a unique feature of this dataset compared to other large river chemistry datasets that allows for estimation of loads and assessment of the role of hydrology in controlling stream Si dynamics. A shapefile delineating polygons for all watersheds is included along with the chemistry database. We also provide summarized watershed scale data including land use/land cover, lithology and soils, and climate variables for all these stream locations generated from globally consistent data sources. The database was constructed to be transparent and reproducible. We provide R code (https://github.com/lter/lterwg-silica-data) and additional reference files used to format and combine data sources as an appendix.

## Methods

### Data acquisition

#### Water chemistry and discharge

We acquired river chemistry and discharge data from published and/or publicly available datasets and through direct requests to researchers or agencies (Supplemental Table 1). Acquired river chemistry data include dissolved Si (DSi), dissolved inorganic N (DIN) and dissolved inorganic P (DIP) concentration data. DIN data include values for NH_4_, NO_3_, or NO_x_. Not all sites reported the same forms of DIN, however, and thus we report data for whichever form(s) were provided in the original dataset (i.e., individual species not a total DIN value). Dissolved inorganic P is reported as “DIP” in this dataset but included data that were originally reported either as soluble reactive phosphorus (SRP) or phosphate (PO_4_).

**Fig. 3:**
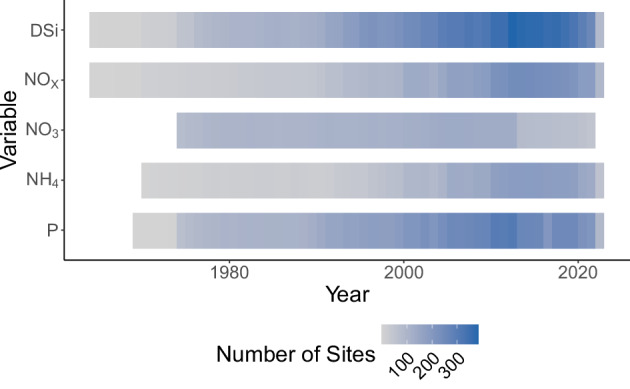
Distribution of chemistry data by solute, period of record, and number of sites.

There are a total of 421 individual sites in the dataset, which all include DSi and river discharge data. 397 sites reported NO_3_ or NO_x_ data, 196 sites report NH_4_, and 339 sites report P (Fig. 3).

Sites spanned 11 Koeppen-Geiger climate zones between −77S and 70 N^25,26^, varied in drainage area from <1 km^2^ to nearly 4 million km^2^, and in mean river discharge from <0.01 m^3^ s^−1^ to nearly 200,000 m^3^ s^−1^ indicating a wide range in catchment conditions included in the dataset.

We established several criteria for including data. Each site was required to have records of daily discharge and discrete observations of dissolved silicon (DSi). We included rivers in the dataset that had a minimum of four years of data, with the period of record for rivers ranging from four to 55 years. The number of observations per year for all stream-variable combinations ranged from 1 to 178 with a median number of observations per year per stream of 14.2 and range of 2.4 to 64. Thus, some years in a dataset for a given stream did have just a single observation, but this was never the case for an entire dataset. Data were required to meet the quality assurance requirements specified by the original data source (see below for additional QA/QC information and technical validation of data). All chemistry samples were derived from “regular” samples (i.e., not field or lab duplicates).

In the process of harmonization, we addressed several additional data quality issues including below detection limit and unreasonable values. DSi data are generally robust, high-quality data because of the consistency of the methods involved and the stability of dissolved Si in filtered water samples. In addition, the concentrations are usually greater than 1 mg Si/L, and thus the problems that can influence measurements of N and P at low concentrations did not occur as frequently.

Stream discharge data were required to have daily values and were all converted to cubic meters per second. Where there were gaps of less than 30 days, we linearly interpolated values and included a field in the data file to indicate whether data are measured or interpolated. Interpolated data represent less than 0.01% of the total dataset.

#### Watershed and climate characteristics

**Fig. 4:**
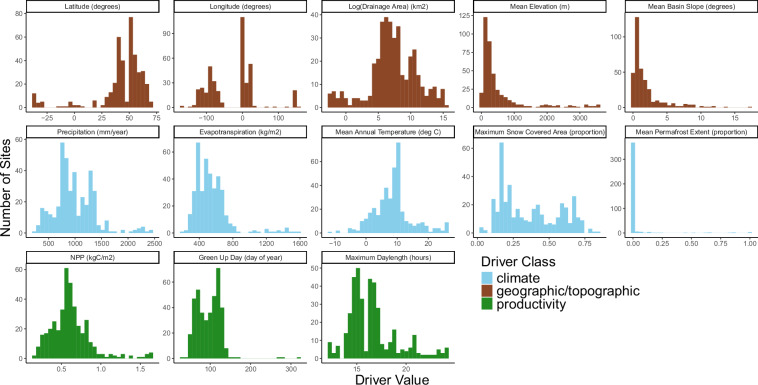
Distribution of watershed characteristics across all the sites included in the dataset, colored by their class (climate, geographic/topographic, productivity). Values shown are mean values for each site over their full period of record.

To characterize climate, ecosystem productivity, land cover, and lithology of contributing watersheds we acquired data from globally available modeled and remotely sensed data sources (Supplemental Table 2, Fig. 4). Specifically, we acquired precipitation, air temperature, snow-covered area, elevation, soil order, land cover, lithology, evapotranspiration, net primary productivity, green-up day, and permafrost. We used spatial data layers with global coverage to have consistent data sources across the extent of our dataset. The spatial resolution of these data sources (Supplemental Table 2) was typically coarser than it might be if we used locally available data sources but had the benefit of providing data generated using a globally-consistent methodology.

In the process of gathering spatial data from grid-based data sources, the initial step involved the procurement or creation of watershed delineations. Where feasible, we sourced existing watershed boundary shapefiles (referenced in Table S3 Johnson *et al*.^22^). For six sites where the original data provider did not supply watershed shapefiles, the HydroBASINS database, as described by Lehner and Grill^27^ was used to construct the necessary watershed delineations. HydroBASINS offers a comprehensive global network of hierarchically organized sub-basins across various scales. In its most detailed sub-basin segmentation, HydroBASINS separates a basin into two smaller sub-basins at junctures where two tributaries converge, each with a minimum upstream area of 100 km². For the compilation of watersheds pertinent to this dataset, the initial step was to pinpoint the basin that overlapped with the sampling coordinates at the most detailed HydroBASINS segmentation level, followed by the successive inclusion of all connected upstream basins. After delineating all relevant basin polygons, they were amalgamated into a unified shapefile, which then served as the definitive boundary for the watershed. Given that the smallest HydroBASINS delineations average around 100 km², which is considerably larger than some of the streams in our study, this method was not applied to basins less than 2000 km² in size.

Data were available at different spatial and temporal resolutions (Supplemental Table 2). For time-varying datasets, we primarily summarized data at an annual scale as that was most commonly available time step across datasets. Surface air temperature, precipitation, green-up day, net primary productivity, and land cover were all available as annual mean values. Evapotranspiration data were available on an 8-day time step, which we summarized to annual mean values. Snow-covered area (percent of watershed covered by snow) was also available at a daily time step, from which we extracted the maximum annual value for use in our models. The number of snow-covered days were generated by multiplying the proportion of snow-covered pixels within a watershed (binary) by the number of days those pixels were snow-covered. For example, a watershed with 10 snow-covered days could have snow cover in 100% of its pixels for 10 days within a year or have only 50% of snow cover for 20 days. This metric was highly correlated with the maximum snow-covered area. We included global land cover data at 30 m resolution^28^. Land cover was available for 1985, 1990, 1995, and annually from 2000–2022. Years between each five-year increment (e.g., 1985–1990) were linearly interpolated. Land cover classes were lumped into forest, grassland and shrubland, wetland and march, tidal wetland, cropland, impervious, ice and snow, water, salt water, and bare (Table S3), and the proportion of each land cover class was reported for each watershed.

The lithology, permafrost, watershed elevation and slope data included in the database were all static values (not time varying). Lithology data were sourced from the PANGEA dataset and lumped into volcanic, sedimentary, plutonic, metamorphic, and carbonate/evaporite. Land cover and lithology categories were further refined as shown in Supplemental Tables 3 & 4. Watershed elevation was measured with the digital elevation model available on WorldClim, which is derived from the 30 second SRTM digital elevation data. Within each watershed boundary the mean, median, minimum, and maximum elevations were all calculated as well as the same summary statistics for basin slope. Permafrost probability was reported as a value between 0 and 1 and represents probability of continuous, discontinuous or sporadic permafrost^29^.

We also include the watershed delineations for nearly all the rivers included in this dataset. This enables future users to gather additional datasets that may provide other variables at finer temporal or spatial resolutions.

### Data harmonization

We built the chemistry and discharge datasets with the intention of easy integration of future additional datasets. The workflow was designed to be flexible enough to ingest datasets in many formats (i.e., wide, long, different column names, units) and to produce a single harmonized datafile with all data with the same units, date formats, variables, and column names.

**Fig. 5:**
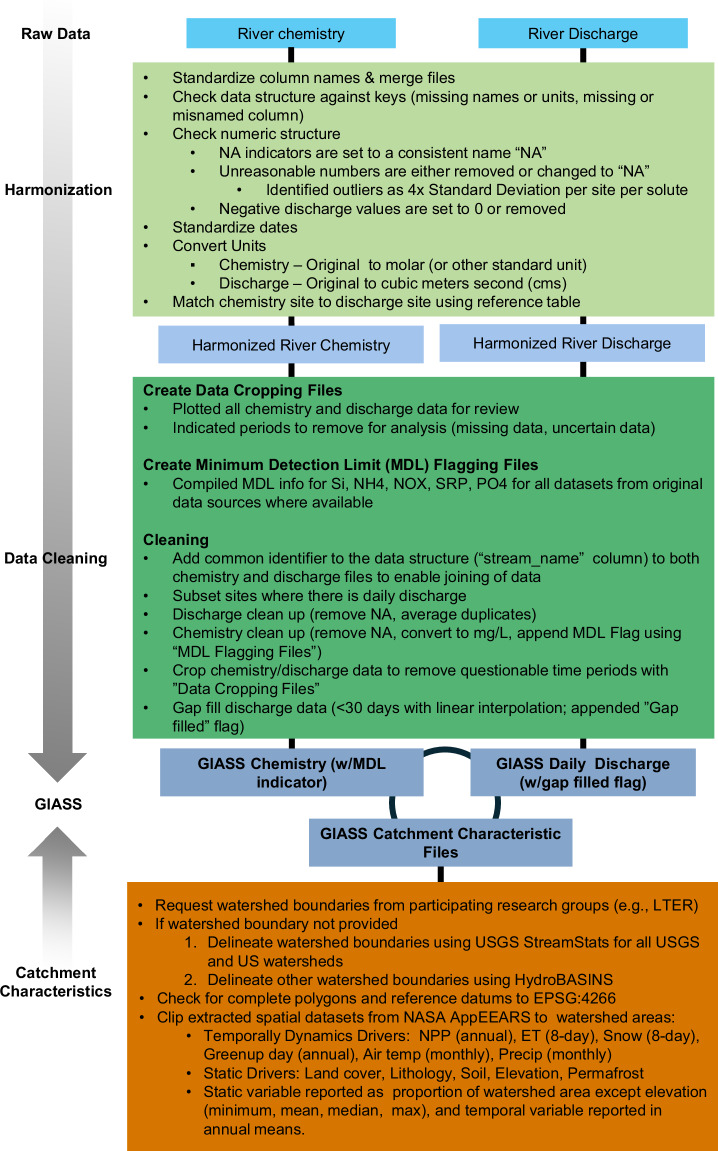
Process by which chemistry, discharge, and spatial data were standardized, reviewed, and harmonized.

All original data sources are listed in Supplemental Table 1 and the harmonization process is shown in Fig. 5. Harmonization included several QA and validation steps for both the chemistry and discharge datasets. These steps included reviewing for missing or unreasonable data values, screening and removing extreme outliers, standardizing date formats, converting units, removing duplicate values or site records and appending minimum detection limit (MDL) flags to chemistry data and gap-filled indicators to the discharge dataset. Additional reference files used to assign MDL values and select periods of the original time series (see Fig. 5) are included as Supplemental Material.

## Data Records

All datasets are located in the U.S. Geological Survey ScienceBase respository^30^.

### Record 1

GlASS chemistry (Si, DIN, and DIP).

This dataset contains 421 sites from 24 different observation networks. Across all sites, periods of records for DSi, DIN, and DIP were 1964–2023, 1964–2023, and 1969–2023, respectively. For all variables across all sites, the mean number of samples per site per year was 14.2. The chemistry dataset is formatted in long format and contains all observations of all solutes for all streams across the dataset. The stream chemistry file is named Chemistry_dat_v2.csv.

#### Research_network

Name of the research network that provided data.

#### Stream_name

Name of the stream or stream site.

#### Date

Date of sample collection.

#### Variable

Name of constituent.

#### value_mgL

Concentration of constituent in milligrams per liter. Units are reported as concentration of Si, N or P. Specifically, DSi as Si; DIN as NO_3_-N, NO_x_-N, NH_x_-N; and either SRP or PO4-P as DIP).

#### remarks

code indicating whether the value is at or below the detection limit (“<”).

### Record 2

GlASS discharge datasets.

The discharge period of record ranged from 1963–2023 The discharge dataset is stored in Discharge_dat.csv and contains daily flow values for all rivers included in the dataset. Where discharge was not continuous, discharge was gap-filled when the gap was <30 days. The column name “indicator” indicates whether the discharge was gap filled or not.

#### Research_network

Name of the research network that provided data.

#### Stream_Name

Name of the stream or stream site.

#### Date

Date of collection.

#### Discharge

Discharge in cubic meters per second.

#### Indicate

Indicates whether value was measured or interpolated.

### Record 3

Shapefiles.

The shapefile dataset is an aggregation of all individual river shapefiles in coordinate reference EPSG:4396.

### Record 4

Mean watershed and climate data parameters.

This dataset includes latitude, longitude, and long-term mean values of all parameters listed in Supplemental Table 2 for 400 sites in the dataset.

### Record 5

Mean annual watershed and climate data parameters.

This dataset includes latitude, longitude, and long-term mean annual values of air temperature, precipitation, net primary productivity, green-up day, snow cover, and land cover for 400 sites in the dataset (as described in Supplemental Table 2).

## Technical Validation

### Chemistry and discharge data

Several steps were taken to validate the data before, during, and after harmonization. Before harmonization, we acquired metadata describing data quality and limitations from the original data source where possible (e.g., USGS or EDI). We required individual data contributors to review their own data prior to submission and provide data that had been validated and QA’d according to their institution’s protocols. We plotted all chemistry and discharge data to visually review and identify errors, long periods of missing data, or other data problems prior to harmonization with other datasets.

During harmonization, we removed unreasonable numbers (e.g., negative concentrations), extreme outlier values (4 times the standard deviation), and duplicate data records present across multiple data sources. We standardized site names across chemistry and discharge datasets (created unique site ID, “Stream_Name” that is common across datasets).

After data harmonization, we plotted all discharge and chemistry data and visually reviewed them for errors and reasonable ranges of values (e.g., to validate unit conversions were correct). We sent a subset of the data back to the original data contributors to review for validity and consistency. We then compared data to previously published values for sites to validate that means and ranges were similar to known values.

**Fig. 6:**
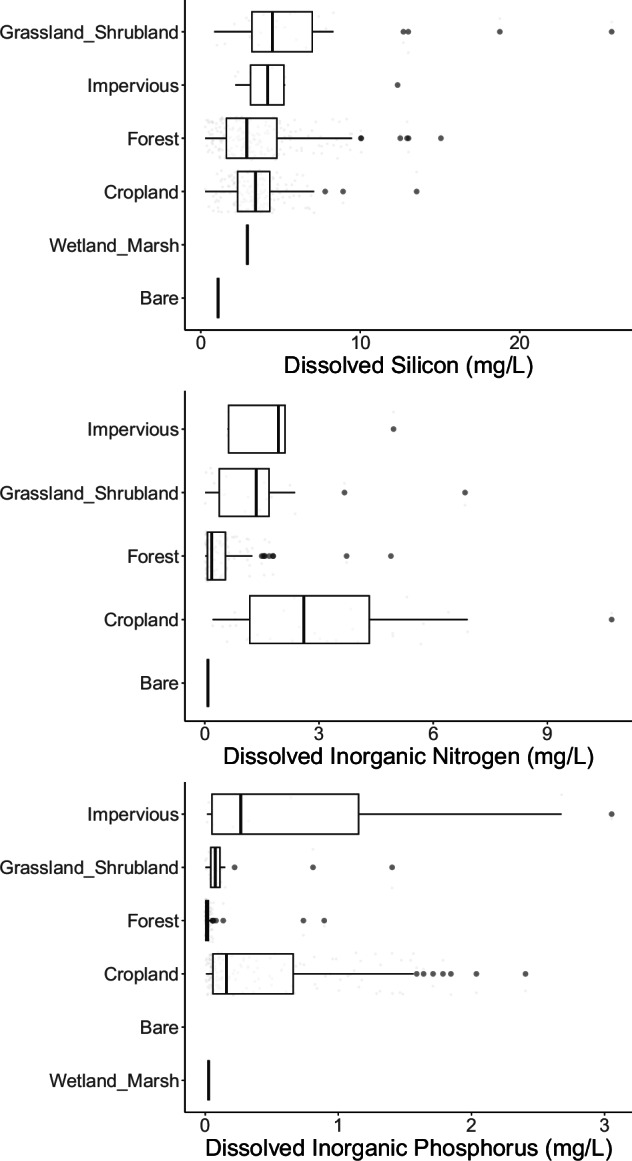
Distribution of (**a**) DSi concentration, (**b**) Dissolved inorganic nitrogen (NO_x_, NO_3_, and NH_4_) and (**c**) dissolved inorganic phosphorus concentration with major land cover. Box plots depict the minimum, first quartile, median, third quartile, and maximum, with outliers depicted as single points.

**Fig. 7:**
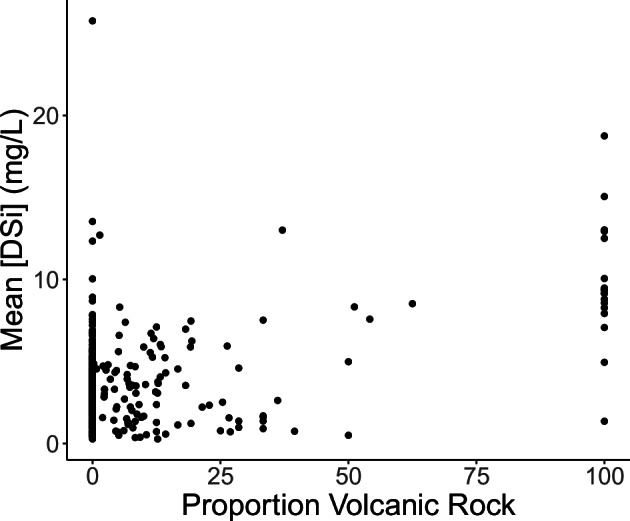
Distribution of DSi concentration with the proportion of volcanic rock in the watershed.

Finally, to ensure our data align with generally understood spatial patterns and mechanistic drivers of river chemistry and discharge, we performed a number of additional comparisons. We evaluated spatial patterns in Si, N, and P concentrations with environmental gradients known to have strong influences on their values (Figs. 6, 7). River Si concentrations typically align well with global distributions of bedrock lithology, increasing with the abundance and weathering rates of silicate rocks^31–33^. That pattern is generally evident in this dataset, which shows the highest concentrations tend to occur in watersheds draining volcanic lithology (Fig. 7). DSi showed less variability with land cover but grassland/shrubland and urban/impervious land highest concentrations on average. River N and P concentrations tend to increase with agricultural and urban land uses^34–36^, which is shown clearly in this dataset as well. Watersheds dominated by cropland, shrubland, and urban land use had the highest concentrations of both inorganic N and P (Fig. 6).

### Spatial data

We largely relied on the original technical validation of the data done by the authors/producers of the data products (Supplemental Tables 1, 2) but did our own evaluation of the values it produced for the watersheds in this dataset.

**Fig. 8:**
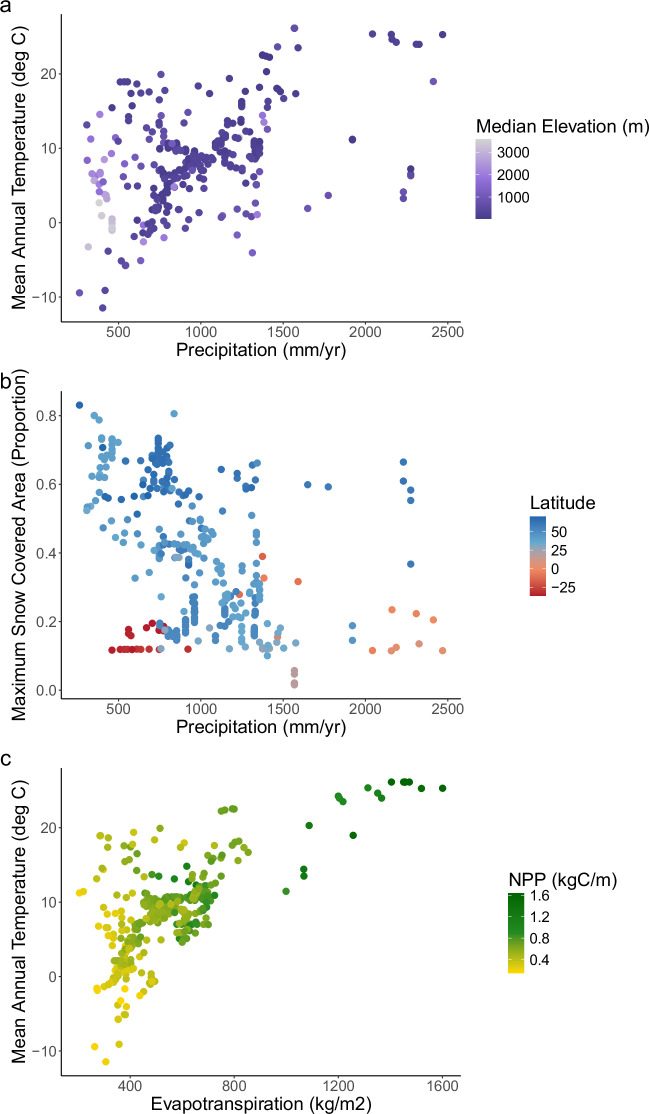
Distributions and relationships among associated watershed characteristics. (**a**) mean annual precipitation, mean annual temperature, and median watershed elevation (Q); (**b**) mean annual precipitation, maximum snow-covered area, and latitude; and (**c**) mean annual evapotranspiration, mean annual temperature, and mean annual NPP (net primary productivity).

Specifically, we reviewed values of the watershed-scale data that were generated to ensure they seemed reasonable and within expected ranges (Figs. 4, 8). For example, we verified that all proportions (e.g., land cover, lithology) added up to 100 percent for each watershed, land cover classes matched our expectations, and neighboring watersheds had similar values to one another for variables such as air temperature and precipitation. After our own internal review, we sent them to site experts to assess whether values were reasonably aligned with known or published values.

In general, we used the data as they were generated from the global products and did not modify the reported values to account for local knowledge or other available data sources. Because we did not have consistent knowledge of all parameters across all sites in our dataset, we did not modify values generated from these data products using other sources of data. We only modified or removed values if they were obviously unreasonable or wrong and largely relied on the internal QA/QC of the satellite products. To account for cases where data products reported values that appeared unlikely, we included quality flags. This was a particular issue for data from the MODIS platform likely as a result of cloud interference and pixel size. MODIS-derived data included snow, evapotranspiration, NPP, and green-up day. The issues were particularly clear for snow data, therefore we included a column that flagged snow data values as “unlikely” (“U”) in cases where snow cover data were reported but the site also had a mean annual temperature > 15 C, latitude < |35| degrees, and elevation < 1000 m. We also included a flag for annual land cover data to indicate greater uncertainty before 2000 because data collection occurred every five years rather than annually as it did after 2000. 

## Usage Notes

### Limitations

#### Stream chemistry

We included the most continuous record available for stream chemistry, but some datasets have large gaps in time, change from reporting one constituent to another (e.g., early data reported as NO_3_ but later data reported as NO_x_) or do not extend to recent years. There are some datasets that have many values at or below the detection limit. We included a remark code to indicate that a value was below detection for a given site and constituent combination, but we did not include the actual minimum detection limit value or replace any values. The stream chemistry dataset includes dissolved forms of nutrients as those were the most frequently available across sites. There are known limitations when using concentrations of these nutrients to describe their availability, as they may not contain the entire pool available for uptake^37–39^ and low concentrations may in fact reflect high biological uptake. Integrating other datasets would be necessary to understand those types of dynamics.

There are other global databases that provide extensive stream chemistry data, including DSi^40–42^. We reviewed these datasets and included some of the available data. Many of them did not have paired stream discharge data so were not included in this product.

#### Stream discharge

Discharge is provided as daily values but some of the values were interpolated. In some cases, longer records existed for discharge, but we did not include data far outside of the chemistry record as this dataset was intended for use in modeling nutrient fluxes not evaluating long-term changes in hydrology.

#### Watershed characteristics

There are some important limitations in the use of the watershed characteristics dataset. Given that the average size of the pixels of the original data products are large relative to the size of some individual watersheds, these data are best used to compare across watersheds at a global scale to capture large-scale environmental gradients and are not well suited to compare across small watersheds (i.e., watersheds smaller than the footprint of the pixel). In addition, the snow, ET, NPP, and green-up day values are generated using the MODIS platform, which does not perform as well in watersheds that experience a lot of cloud cover (e.g., tropical watersheds). As stated above, data flags were added to clarify where values generated by global products were uncertain or unlikely.

## Supplementary information

Supplementary Information File

## Data Availability

All data are available at Global Aggregation of Stream Silica (GlASS) (ver. 2.0, July 2025) - ScienceBase-Catalog.

## Code availability

Code used to clean and harmonize dataset is located here: https://github.com/lter/lterwg-silica-data. This repository includes scripts that were used to process and clean the original data sources cited in Supplemental Table 1, specifically 00-harmonize_chemistry.R and 00_harmonize_discharge.R. Those harmonized data files were then further modified to produce the chemistry and discharge data described here using 01-wrtds-step02-wrangling.R. Additional scripts are included in the repository to process data through the Weighted Regression on Time, Discharge and Season model^43^ (Hirsch *et al*. 2010).
