# Supplementary material for: GlASS - Global Aggregation of Stream Silica
Source: Sci Data. 2025 Oct 20;12:1658. doi: 10.1038/s41597-025-05937-2 (PMC12537932; doi:10.1038/s41597-025-05937-2)
Supplement: Supplementary file 1 — Supplementary Information File [file 41597_2025_5937_MOESM1_ESM.docx]

Supplemental Material

**Supplemental Table 1 -** Research network type, research site name and abbreviation, chemistry data source, and discharge data source information for data included in this data product. Research site abbreviation is how each research network is identified in the published dataset. LTER = U.S. National Science Foundation Long-term Ecological Research Network. USGS = United States Geological Survey. Acronyms or short dataset names are provided that match “Research Network” column of the dataset. This table is adapted and expanded from Table S1 of Johnson et al. 2024^1^.

| **Research Network Type** | **Research Site Name (Acronym)** | **Chemistry Data Source** | **Discharge Data Source** |
| --- | --- | --- | --- |
| LTER | Toolik Field Station (ARC) | Kling, G. 2022. Biogeochemistry data set for soil waters, streams, and lakes near Toolik Lake on the North Slope of Alaska, 2012 through 2020 ver 2. Environmental Data Initiative. https://doi.org/10.6073/pasta/4e25db9ae9372f5339f2795792814845 (Accessed 2022-12-20).    Kling, G. 2019. Biogeochemistry data set for Imnavait Creek Weir on the North Slope of Alaska 2002-2018 ver 9. Environmental Data Initiative. https://doi.org/10.6073/pasta/733c73c6ebffeaec6970b2b0f4dddfe6 (Accessed 2022-12-20). | Arp C., and Stuefer S.L., 2017. Hydrological and meteorological data from the North Slope of Alaska. University of Alaska Fairbanks, Water and Environmental Research Center. http://ine.uaf.edu/werc/werc-projects/teon/current-stations/franklin-bluffs/. Accessed April 17, 2017.    https://arc-lter.ecosystems.mbl.edu/landscape-interactions/landscape-interactions-discharge |
| LTER | Martinelli - Niwot Ridge (NWT) | Caine, T. 2021. Stream water chemistry data for Martinelli basin, 1984 - ongoing. ver 4. Environmental Data Initiative. https://doi.org/10.6073/pasta/5534a25d64cd018f9c96a6a4c2f49315 (Accessed 2022-12-20). | Caine, T., J. Morse, and Niwot Ridge LTER. 2022. Streamflow for Martinelli basin, 1982 - ongoing. ver 14. Environmental Data Initiative. https://doi.org/10.6073/pasta/76fffe560f896e1e9a04ab34c114b8a5 (Accessed 2022-12-20). |
| LTER | Saddle Stream - Niwot Ridge (NWT) | Williams, M. 2021. Stream water chemistry data for Saddle stream (007), 1994 - ongoing. ver 4. Environmental Data Initiative. https://doi.org/10.6073/pasta/d8a48465249f41936816a5d0d1d22e87 (Accessed 2022-12-20). | Caine, T., J. Morse, and Niwot Ridge LTER. 2022. Streamflow data for Saddle stream, 1999 - ongoing. ver 7. Environmental Data Initiative. https://doi.org/10.6073/pasta/3abf11e44afab0e8605684549f3868a3 (Accessed 2022-12-20). |
| LTER | Albion - Niwot Ridge (NWT) | Caine, T. 2021. Stream water chemistry data for Albion site, 1982 - ongoing. ver 14. Environmental Data Initiative. https://doi.org/10.6073/pasta/59396e2b8d291badcfb140425e1b3a38 (Accessed 2022-12-20). | Caine, N., J. Morse, and Niwot Ridge LTER. 2022. Streamflow data for Albion camp, 1981 - ongoing. ver 17. Environmental Data Initiative. https://doi.org/10.6073/pasta/81ef15564db3a999ea28c02697550525 (Accessed 2022-12-20). |
| LTER | McMurdo Dry Valleys (MCM) | Gooseff, M. and W. Lyons. 2022. Ion concentrations in glacial meltwater streams, McMurdo Dry Valleys, Antarctica (1993-2020, ongoing) ver 13. Environmental Data Initiative. <https://doi.org/10.6073/pasta/275ee580f3c93f077dd7ddcce1f2ecdd> (Accessed 2024-08-30). | Gooseff, M. and D. McKnight. 2024. Daily summarized seasonal measurements of discharge, water temperature, and specific conductivity from Andersen Creek at H1, McMurdo Dry Valleys, Antarctica (1993-2023, ongoing) ver 6. Environmental Data Initiative. https://doi.org/10.6073/pasta/737849f3e573ae253e0bc066bfac439d (Accessed 2024-08-30).  Gooseff, M. and D. McKnight. 2024. Daily summarized seasonal measurements of discharge, water temperature, and specific conductivity from Lawson Creek at B3, McMurdo Dry Valleys, Antarctica (1994-2023, ongoing) ver 9. Environmental Data Initiative. https://doi.org/10.6073/pasta/ea86ba852f77355e97fc4d063c220601 (Accessed 2024-08-30).  Gooseff, M. and D. McKnight. 2024. Daily summarized seasonal measurements of discharge, water temperature, and specific conductivity from Green Creek at F9, McMurdo Dry Valleys, Antarctica (1990-2023, ongoing) ver 5. Environmental Data Initiative. https://doi.org/10.6073/pasta/211b0705c5e89ab1b8ea7fb908432a81 (Accessed 2024-08-30).  Gooseff, M. and D. McKnight. 2021. Daily summarized seasonal measurements of discharge, water temperature, and specific conductivity from Canada Stream at F1, McMurdo Dry Valleys, Antarctica (1990-2020, ongoing) ver 5. Environmental Data Initiative. https://doi.org/10.6073/pasta/84d7b8951eba8d4045c9811bf22eb012 (Accessed 2022-12-20).  McKnight, D. and M. Gooseff. 2022. Daily summarized seasonal measurements of discharge, water temperature, and specific conductivity from Priscu Stream at B1, McMurdo Dry Valleys, Antarctica (1993-2011) ver 3. Environmental Data Initiative. https://doi.org/10.6073/pasta/225599878869bdc7fb98def1fb50db4e (Accessed 2022-12-20).  Gooseff, M. and D. McKnight. 2024. Daily summarized seasonal measurements of discharge, water temperature, and specific conductivity from Von Guerard Stream at F6, McMurdo Dry Valleys, Antarctica (1990-2023, ongoing) ver 5. Environmental Data Initiative. https://doi.org/10.6073/pasta/a7211822a5a79de9ee2b695c9a3f7ab9 (Accessed 2024-08-30)  Gooseff, M. and D. McKnight. 2024. Daily summarized seasonal measurements of discharge, water temperature, and specific conductivity from the Onyx River at Lake Vanda, McMurdo Dry Valleys, Antarctica (1969-2023, ongoing) ver 6. Environmental Data Initiative. https://doi.org/10.6073/pasta/59da08b89924a43730a1bb5a5913df73 (Accessed 2024-08-30).  Gooseff, M. and D. McKnight. 2024. Daily summarized seasonal measurements of discharge, water temperature, and specific conductivity from the Onyx River at Lower Wright, McMurdo Dry Valleys, Antarctica (1972-2023, ongoing) ver 3. Environmental Data Initiative. https://doi.org/10.6073/pasta/68c483362ba88faf3e8d681f1deee620 (Accessed 2024-08-30).  Gooseff, M. and D. McKnight. 2024. Daily summarized seasonal measurements of discharge, water temperature, and specific conductivity from Harnish Creek at F7, McMurdo Dry Valleys, Antarctica (2001-2023, ongoing) ver 4. Environmental Data Initiative. https://doi.org/10.6073/pasta/88ca71b6c35aba4bc8413dbb35d6589f (Accessed 2024-08-30).  Gooseff, M. and D. McKnight. 2024. Daily summarized seasonal measurements of discharge, water temperature, and specific conductivity from Commonwealth Stream at C1, McMurdo Dry Valleys, Antarctica (1993-2023, ongoing) ver 26. Environmental Data Initiative. https://doi.org/10.6073/pasta/488ba9c31560d4498f7d3270dd514e38 (Accessed 2024-08-30).  Gooseff, M. and D. McKnight. 2024. Daily summarized seasonal measurements of discharge, water temperature, and specific conductivity from Delta Stream at F10, McMurdo Dry Valleys, Antarctica (1990-2023, ongoing) ver 5. Environmental Data Initiative. https://doi.org/10.6073/pasta/818d5546a1b3ca7321bbb6231d118bd7 (Accessed 2024-08-30).  Gooseff, M. and D. McKnight. 2024. Daily summarized seasonal measurements of discharge, water temperature, and specific conductivity from Crescent Stream at F8, McMurdo Dry Valleys, Antarctica (1990-2023, ongoing) ver 5. Environmental Data Initiative. https://doi.org/10.6073/pasta/a045277c6671c3bc27f9d654b7f9de0c (Accessed 2024-08-30). |
|  |  |  |  |
| LTER | Hubbard Brook Experimental Forest (HBR) | Hubbard Brook Watershed Ecosystem Record (HBWatER). 2022. Continuous precipitation and stream chemistry data, Hubbard Brook Ecosystem Study, 1963 – present. ver 8. Environmental Data Initiative. https://doi.org/10.6073/pasta/5e9d1771f114913c2ca8c98520c230ad (Accessed 2022-12-20). | USDA Forest Service, Northern Research Station. 2022. Hubbard Brook Experimental Forest: Daily Streamflow by Watershed, 1956 - present ver 12. Environmental Data Initiative. https://doi.org/10.6073/pasta/15b300e96c2d2f9785d0155b3e18b0e9 (Accessed 2022-12-20). |
| LTER | H.J. Andrews Experimental Forest (AND) | http://andlter.forestry.oregonstate.edu/data/abstract.aspx?dbcode=CF002 | http://andlter.forestry.oregonstate.edu/data/abstract.aspx?dbcode=HF004 |
| LTER | Luquillo (LUQ) | McDowell, W. and International Institute of Tropical Forestry(IITF), USDA Forest Service.. 2022. Chemistry of stream water from the Luquillo Mountains ver 4923061. Environmental Data Initiative. https://doi.org/10.6073/pasta/570231c2807a6396ced6a89ef7547bd4. | Leon, M. 2022. USGS Long-term daily streamflow data at several LEF locations ver 1315151. Environmental Data Initiative. https://doi.org/10.6073/pasta/a87891ff406503c740b65067d62cbf61. |
| Research Institute | Krycklan Catchment (Krycklan) | [SITES database](https://data.fieldsites.se/portal/#%7B%22filterCategories%22%3A%7B%22station%22%3A%5B%22Svartberget%22%5D%7D%7D) | Laudon, H, Lidberg, W., Sponseller, R.A., Hasselquist, E.M., Westphal, F., Östlund, L., Sandström, C., Järveoja, J., Peichl, M., Ågren, A.M. (2022). Emerging technology can guide ecosystem restoration for future water security. *Hydrological Processes, DOI: 10.1002/hyp.14729*. |
| Research Institute | Arctic Great Rivers Observatory (GRO) | [Data - Arctic Great Rivers](https://arcticgreatrivers.org/data/) | [Data - Arctic Great Rivers](https://arcticgreatrivers.org/data/) |
| Research Network | GLORICH - Germany | Hartmann, Jens; Lauerwald, Ronny; Moosdorf, Nils (2019): GLORICH - Global river chemistry database [dataset]. PANGAEA, https://doi.org/10.1594/PANGAEA.902360 | https://www.fgg-elbe.de/elbe-datenportal-en.html |
| Research Network | GLORICH - Cameroon | Hartmann, Jens; Lauerwald, Ronny; Moosdorf, Nils (2019): GLORICH - Global river chemistry database [dataset]. PANGAEA, <https://doi.org/10.1594/PANGAEA.902360>;  Viers, J., B. Dupre, J. Braun, S. Deberdt, B. Angeletti, J.N. Ngoupayou, and A. Michard. 2000. Major and trace element abundances, and strontium isotopes in the Nyong basin rivers (Cameroon): constraints on chemical weathering processes and elements transport mechanisms in humid tropical environments. Chemical Geology 169(1-2): 211-241. <https://doi.org/10.1016/S0009-2541(00)00298-9> | [https://theses.fr/1997PA066482](https://gcc02.safelinks.protection.outlook.com/?url=https%3A%2F%2Ftheses.fr%2F1997PA066482&data=05%7C02%7Ckjankowski%40usgs.gov%7C192adc11781542c5816708dd6e31a865%7C0693b5ba4b184d7b9341f32f400a5494%7C0%7C0%7C638787881736451475%7CUnknown%7CTWFpbGZsb3d8eyJFbXB0eU1hcGkiOnRydWUsIlYiOiIwLjAuMDAwMCIsIlAiOiJXaW4zMiIsIkFOIjoiTWFpbCIsIldUIjoyfQ%3D%3D%7C0%7C%7C%7C&sdata=qk2bTMwELeLft%2Bml3YxhpnlCeCi92Tl6ml82RKJXl7A%3D&reserved=0) |
| Research Network | HYdro-geochemistry of the AMazonian Basin (HYBAM) | [SO-HyBam – Service d'observation des ressources en eaux du bassin de l'Amazone](https://hybam.obs-mip.fr/) | [SO-HyBam – Service d'observation des ressources en eaux du bassin de l'Amazone](https://hybam.obs-mip.fr/) |
| National | Finnish Environment Institute | Finnish data were made available through the Finnish Environmental Institute (compiled from https://www.avoindata.fi/en by P. Kortelainen and A. Räike). | Finnish data were made available through the Finnish Environmental Institute (compiled from https://www.avoindata.fi/en by P. Kortelainen and A. Räike). |
| National | Norwegian River Monitoring Programme (NIVA) | Chemistry data is available from the Norwegian Environment Agency’s data portal: [https://vannmiljo.miljodirektoratet.no](https://nam04.safelinks.protection.outlook.com/?url=https%3A%2F%2Fvannmiljo.miljodirektoratet.no%2F&data=05%7C01%7Cjohnkeir%40oregonstate.edu%7Cd8563d3ca84948c0dd5308dbd3c48c52%7Cce6d05e13c5e4d6287a84c4a2713c113%7C0%7C0%7C638336613345201209%7CUnknown%7CTWFpbGZsb3d8eyJWIjoiMC4wLjAwMDAiLCJQIjoiV2luMzIiLCJBTiI6Ik1haWwiLCJXVCI6Mn0%3D%7C3000%7C%7C%7C&sdata=yqdGRTIePmgW2OI7PyYKugrD4wysOYMzxp6MKJX4bhQ%3D&reserved=0). Data were made available through the Norwegian River Monitoring Programme (compiled by Ø. Kaste). | Discharge data is available on the Norwegian Water Resource and Energy Directorate Website: [https://sildre.nve.no/](https://nam04.safelinks.protection.outlook.com/?url=https%3A%2F%2Fsildre.nve.no%2F&data=05%7C01%7Cjohnkeir%40oregonstate.edu%7Cd8563d3ca84948c0dd5308dbd3c48c52%7Cce6d05e13c5e4d6287a84c4a2713c113%7C0%7C0%7C638336613345201209%7CUnknown%7CTWFpbGZsb3d8eyJWIjoiMC4wLjAwMDAiLCJQIjoiV2luMzIiLCJBTiI6Ik1haWwiLCJXVCI6Mn0%3D%7C3000%7C%7C%7C&sdata=I%2FsYYjeaoQizWxWlTFmJqGXMEraeYZfuDcR1Lmn5Xao%3D&reserved=0)  Data were made available through the Norwegian River Monitoring Programme (compiled by Ø. Kaste). |
| National | Swedish Government Monitoring Program | [Department of Aquatic Sciences and Assessment \| The external web (slu.se)](https://www.slu.se/vatten-miljo/) | [Water web \| SMHI](https://www.smhi.se/data/hydrologi/vattenwebb) |
| National | Camels-CHEM (USGS) | Sterle, G., Perdrial, J., D.W. Kincaid, K.L. Underwood, D.M. Rizzo, I.U. Haq, Li, L., B.S. Lee, T. Adler, H. Wen, H. Middleton, and A.A. Harpold: CAMELS-Chem: Augmenting CAMELS (Catchment Attributes and Meteorology for Large-sample Studies) with Atmospheric and Stream Water Chemistry Data, HESS, 28, 611-630; https://doi.org/10.5194/hess-28-611-2024. | [USGS Water Data for the Nation](https://doi.org/10.5066/F7P55KJN) |
| National | National Water Quality Network (USGS) | Casey Lee, 2022, Nutrient and pesticide data collected from the USGS National Water Quality Network and previous networks, 1950-2021: U.S. Geological Survey, https://doi.org/10.5066/P948Z0VZ | [USGS Water Data for the Nation](https://doi.org/10.5066/F7P55KJN) |
| National | Upper Mississippi River Restoration Program (UMR) | [Upper Midwest Environmental Sciences Center - Water Quality Data (usgs.gov)](https://umesc.usgs.gov/data_library/water_quality/water_quality_data_page.html) | [USGS Water Data for the Nation](https://doi.org/10.5066/F7P55KJN) |
| National | Sagehen Creek Observatory (Sagehen) | [SAGEHEN C NR TRUCKEE CA - USGS Water Data for the Nation](https://waterdata.usgs.gov/monitoring-location/10343500/#parameterCode=00065&period=P7D) | [USGS Water Data for the Nation](https://doi.org/10.5066/F7P55KJN) |
| National | United Kingdom National Flow Archive (UK) | Compiled from National River Flow Archive - [National River Flow Archive \| National River Flow Archive](https://nrfa.ceh.ac.uk/) | Compiled from National River Flow Archive - [National River Flow Archive \| National River Flow Archive](https://nrfa.ceh.ac.uk/) |
| National Laboratory Test Site | Walker Branch | Mulholland, P.J., and N.A. Griffiths. 2016. Walker Branch Watershed: Weekly Stream Water Chemistry. Carbon Dioxide Information Analysis Center, Oak Ridge National Laboratory, U.S. Department of Energy, Oak Ridge, Tennessee, U.S.A. http://dx.doi.org/10.3334/CDIAC/ornlsfa.009 | Mulholland, P.J., and N.A. Griffiths. 2016. Walker Branch Watershed: 15-minute and Daily Stream Discharge and Annual Runoff. Carbon Dioxide Information Analysis Center, Oak Ridge National Laboratory, U.S. Department of Energy, Oak Ridge, Tennessee, U.S.A.<http://dx.doi.org/10.3334/CDIAC/ornlsfa.007> |
| University | Lamprey River Hydrologic Observatory (LMP) | [Data and Maps \| NH Water Resources Research Center (WRRC) (unh.edu)](https://wrrc.unh.edu/data-and-maps); Wymore, AS, MD Shattuck, JD Potter, L Snyder, and WH McDowell. 2021. The Lamprey River Hydrological Observatory: suburbanization and changing seasonality. Hydrological Processes. doi: 10.1002/hyp.14131 | [LAMPREY RIVER NEAR NEWMARKET, NH - USGS Water Data for the Nation](https://waterdata.usgs.gov/monitoring-location/01073500/) |
| State | South Florida Management District (KRR) | DBHYDRO; www.sfwmd/dbhydro | DBHYDRO; www.sfwmd/dbhydro |
| Provincial | Canadian Provincial Monitoring (Canada) | [National Long-term Water Quality Monitoring Data - ECCC Data Catalogue](https://data-donnees.az.ec.gc.ca/data/substances/monitor/national-long-term-water-quality-monitoring-data/) | [Water Survey of Canada - Canada.ca](https://www.canada.ca/en/environment-climate-change/services/water-overview/quantity/monitoring/survey.html) |
| State | Australian state monitoring networks | Lintern, A., S. Liu, C. Minaudo, R. Dupas, D. Guo, K. Zhang, U. Bend-Michl, and C. Duvert. 2021. The influence of climate on water chemistry states and dynamics in rivers across Australia. Hydrological Processes 35(21); <https://doi.org/10.1002/hyp.14423> | Lintern, A., S. Liu, C. Minaudo, R. Dupas, D. Guo, K. Zhang, U. Bend-Michl, and C. Duvert. 2021. The influence of climate on water chemistry states and dynamics in rivers across Australia. Hydrological Processes 35(21); <https://doi.org/10.1002/hyp.14423> |
| State | Murray-Darling (MD) | Biswas, T.K. and L.M. Mosley. 2018. From mountain ranges to sweeping plans, in droughts and flooding rains; River Murray water quality over the last four decades. Water Resources Management 33: 1087-1101. <https://link.springer.com/article/10.1007/s11269-018-2168-1> | https://riverdata.mdba.gov.au/ |

**Supplemental Table 2 -** Description, source and spatial/temporal resolution watershed spatial data.

| **Parameter** | **Data Source** | **Units** | **Spatial Resolution** | **Temporal Resolution** |
| --- | --- | --- | --- | --- |
| Air Temperature | Global Historical Climatology Network and Climate Anomaly Monitoring System (GHCN_CAMS) Gridded 2m Temperature (Land); https://psl.noaa.gov/data/gridded/data.ghcncams.html | degrees Celsius | 0.5 degrees | Annual, 1948-2022 |
| Precipitation | National Center for Environmental Information Global Precipitation Climatology Project Monthly Precipitation Data Record; doi:10.7289/V56971M6 | mm / day | 2.5 degrees | Annual, 1979-2022 |
| Climate Zone | Data derived from: Rubel and Kottek (2010): [doi:10.1127/0941-2948/2010/0430](https://doi.org/10.1127%2F0941-2948%2F2010%2F0430); Applied using kgc R package (Bryant et al. (2017)) | NA | ¼ degree | Static |
| Evapotranspiration | Running, S., Mu, Q., Zhao, M., Moreno, A. (2021). *MODIS/Terra Net Evapotranspiration Gap-Filled 8-Day L4 Global 500m SIN Grid V061*. NASA EOSDIS Land Processes Distributed Active Archive Center. Accessed 2023-11-01 from <https://doi.org/10.5067/MODIS/MOD16A2GF.061>. Accessed November 1, 2023. | kg / m^2^ | 500 meter | Annual, 2001-2021 |
| Maximum Snow-Covered Area | MODIS Snow and Ice mapping project 8-Day MOD10A2; https://modis-snow-ice.gsfc.nasa.gov/?c=MOD10A2 | Proportion of watershed | 500 meter | Annual, 2001-2021 |
| Number of days with snow | MODIS Snow and Ice mapping project 8-Day MOD10A2; https://modis-snow-ice.gsfc.nasa.gov/?c=MOD10A2 | Days | 500 meter | Annual, 2001-2021 |
| Green-Up Day | Friedl, M., Gray, J., Sulla-Menashe, D. (2022). *MODIS/Terra+Aqua Land Cover Dynamics Yearly L3 Global 500m SIN Grid V061*. NASA EOSDIS Land Processes Distributed Active Archive Center. Accessed 2023-11-02 from <https://doi.org/10.5067/MODIS/MCD12Q2.061>. | date | 500 meter | Annual, 2001-2019 |
| Net Primary Productivity (NPP) | Running, S., Zhao, M. (2021). *MODIS/Terra Net Primary Production Gap-Filled Yearly L4 Global 500m SIN Grid V061*. NASA EOSDIS Land Processes Distributed Active Archive Center. Accessed 2023-11-02 from <https://doi.org/10.5067/MODIS/MOD17A3HGF.061>. | kgC/m²/year | 500 meter | Annual, 2001-2021 |
| Land Cover | Global 30-meter Land Cover Change Dataset (GLC_FCS30D) | Proportion of watershed | 30 meter | 1985, 1990, 1995, Annual from 2000-2022 |
| Lithology | Global Lithological Map Database v1.0; PANGAEA; https://doi.org/10.1594/PANGAEA.788537 | NA | 0.5 degrees | Static |
| Maximum Daylength | daylength function from R package *chillR* | hours | NA | Static |
| Watershed elevation | WorldClim (derived from SRTM Digital Elevation Model) | meters | 1 kilometer | Static (2000) |
| Watershed slope | WorldClim (derived from SRTM Digital Elevation Model) | meters | 1 kilometer | Static (2000) |
| Soil Order | SoilGrids250m 2017-03 - Predicted WRB 2006 subgroup classes | percent | 250 meter | Static (2006) |
| Permafrost probability | Alfred Wegener Institute, Helmholtz Centre for Polar and Marine Research, PANGAEA; Obu et al. 2018 (<https://doi.org/10.1594/PANGAEA.888600>) | percent | 1 kilometer | Static (2000-2016) |

**Supplemental Table 3.** Land cover classification from original data source, [GLC_FCS30D Global 30-meter Land Cover Change Dataset (1985-2022) - awesome-gee-community-catalog](https://gee-community-catalog.org/projects/glc_fcs/).

| **GLC_FCS30D Land Class** | **Upscaled Land Class** |
| --- | --- |
| Bare_areas | Bare |
| Closed_deciduous_broadleaved_forest | Forest |
| Closed_deciduous_needle_leaved_forest | Forest |
| Closed_evergreen_broadleaved_forest | Forest |
| Closed_evergreen_needle_leaved_forest | Forest |
| Closed_mixed_leaf_forest | Forest |
| Consolidated_bare_areas | Bare |
| Deciduous_shrubland | Grassland_Shrubland |
| Evergreen_shrubland | Grassland_Shrubland |
| Filled_value | Filled_Value |
| Flooded_flat | Wetland_Marsh |
| Grassland | Grassland_Shrubland |
| Herbaceous_cover_cropland | Cropland |
| Impervious_surfaces | Impervious |
| Irrigated_cropland | Cropland |
| Lichens_and_mosses | Bare |
| Mangrove | Tidal_Wetland |
| Marsh | Wetland_Marsh |
| Open_deciduous_broadleaved_forest | Forest |
| Open_deciduous_needle_leaved_forest | Forest |
| Open_evergreen_broadleaved_forest | Forest |
| Open_evergreen_needle_leaved_forest | Forest |
| Open_mixed_leaf_forest | Forest |
| Permanent_ice_and_snow | Ice_Snow |
| Rainfed_cropland | Cropland |
| Saline | Salt_Water |
| Salt_marsh | Tidal_Wetland |
| Shrubland | Grassland_Shrubland |
| Sparse_herbaceous | Grassland_Shrubland |
| Sparse_shrubland | Grassland_Shrubland |
| Sparse_vegetation | Grassland_Shrubland |
| Swamp | Wetland_Marsh |
| Tidal_flat | Tidal_Wetland |
| Tree_or_shrub_cover_cropland | Cropland |
| Unconsolidated_bare_areas | Bare |
| Water_body | Water |

**Supplemental Table 4:** Lithology classification (Table from Johnson et al.^2^)

| **Pangea Lithology** | **Upscaled Lithology** |
| --- | --- |
| unconsolidated sediments | sedimentary |
| siliciclastic sedimentary rocks | sedimentary |
| mixed sedimentary rocks | sedimentary |
| carbonate sedimentary rocks | carbonate/evaporite |
| evaporites | carbonate/evaporite |
| metamorphic rocks | metamorphic |
| acid plutonic rocks | plutonic |
| intermediate plutonic rocks | plutonic |
| basic plutonic rocks | plutonic |
| pyroclastic | volcanic |
| acid volcanic rocks | volcanic |
| intermediate volcanic rocks | volcanic |
| basic volcanic rocks | volcanic |

**REFERENCES**

1. Johnson, K. *et al.* Establishing fluvial silicon regimes and their stability across the Northern Hemisphere. *Limnol Oceanogr Letters* **9**, 237–246 (2024).

2. Johnson, K. *et al.* Climate, hydrology, and nutrients control the seasonality of Si concentrations in rivers. *Journal of Geophysical Research: Biogeosciences* **129**, (2024).
